# Exploring the contextual factors, behaviour change techniques, barriers and facilitators of interventions to improve oral health in people with severe mental illness: A qualitative study

**DOI:** 10.3389/fpsyt.2022.971328

**Published:** 2022-10-11

**Authors:** Masuma Pervin Mishu, Mehreen Riaz Faisal, Alexandra Macnamara, Wael Sabbah, Emily Peckham, Liz Newbronner, Simon Gilbody, Lina Gega

**Affiliations:** ^1^Department of Health Sciences, Faculty of Sciences, University of York, Heslington, United Kingdom; ^2^Department of Epidemiology and Public Health, University College London, London, United Kingdom; ^3^Hull York Medical School, University of York, Heslington, United Kingdom; ^4^Faculty of Dentistry, Oral & Craniofacial Sciences, King’s College London, London, United Kingdom

**Keywords:** severe mental illness, oral health intervention, contextual factors, barriers and facilitators, qualitative study, behaviour change techniques

## Abstract

People with severe mental illness (SMI) have significantly poorer oral health compared to people without SMI and interventions targetted to improve oral health in this population failed to show any long-term improvement. Interventions are influenced by many contextual factors ranging from individual to systems level. This study aimed to understand the contextual factors, behaviour change techniques of the available oral health interventions and explore the barriers to and facilitators for engagement with these interventions from the perspectives of people with SMI (service users) and related service providers. Intervention details were extracted from 12 intervention studies identified from a previous systematic review using the template for intervention description and replication checklist (TIDieR) and behaviour change techniques (BCTs) were coded using the behaviour change technique taxonomy v1. Sixteen individual BCTs were identified and out of which “*4.1 instructions on how to perform the behaviour”* (*n* = 9) and *“6.1 demonstration of behaviour”* (*n* = 6) were most frequently used BCTs. Video vignettes prepared from the different intervention components identified from existing studies were shown to service users and service providers in dyadic or one-to-one interview format to elicit their views on barriers and facilitators for engagement with the intervention components. Interviews were analysed using Framework analysis and were guided by theoretical domains framework (TDF); and capability, opportunity and motivation (COM-B) model of behaviour change. Main facilitators identified to increase capability, opportunity and motivation of service users were the involvement of carers/care coordinators and integration of dental and mental health care, provision of oral health/hygiene information/products at an appropriate level and provision of tailored support according to individual needs and preferences. Barriers identified were related to lack of communication skills of the service providers, provision of coordinated care, lack of support in visiting a dentist and navigating the payment system and long follow up times. Appropriate training was considered as a facilitator, and staff turnovers and workload were considered as main barriers by the service providers. The findings suggest that comprehensive interventions that target barriers and enhance facilitators from individual to systems level are needed to improve oral health outcomes of people with SMI.

## Introduction

People with severe mental illness (SMI) comprise between 2 and 4% of the population (1). This population group face significant health inequalities, having poorer health outcomes and a lower life expectancy compared to people without SMI (2). People with SMI also have poorer oral health, for example those with SMI have 3.4 times the odds of having lost all their teeth than the general population. They also have, on average, 6.2 more decayed teeth than those without SMI (3, 4).

Poor oral health has a profound effect on general health and quality of life, and can have an impact on social life, self-esteem and social interactions (5). Oral-health-related quality of life (OHRQoL) is also affected in people with SMI, due to worse oral health outcomes in this population (6). Furthermore, oral diseases are associated with other physical health conditions such as diabetes (7) and coronary heart diseases (8, 9). To tackle this inequality, an effective intervention to improve oral health among people with SMI is essential, and to date, several interventions have been tried for this population.

A Cochrane systematic review exploring the effectiveness of oral health interventions in people with SMI, suggested that an oral health education intervention led to better oral hygiene, but its clinical significance was unclear (10). Furthermore, a recent systematic review investigating such interventions included 12 studies (11) and identified five broad categories of intervention: dental education, motivational interviewing, dental checklist, dietary change and incentives. Despite statistically significant short-term changes in plaque indices and oral health behaviours as a result of interventions using dental education, motivational interviewing and incentives, it is unclear if these changes led to clinically meaningful improvements. Clinically relevant outcomes like “tooth loss” and “oral health related quality of life” (12) were not assessed in these studies. Overall, the review found that most of the interventions were designed to improve oral health via individual level behavioural change. Furthermore, there is a paucity of information on the patient, carer, and service/healthcare level factors that influence oral health improvement in people with SMI. In a narrative review, Slack et al. aimed to identify individual, organisational and systemic levels barriers (13). However, the system and policy factors were not covered in depth in the review partly due to fewer studies focussing on more upstream approaches to improving oral health outcomes in this group. To improve the oral health of people with SMI, it is important to develop and implement effective interventions that are tailored to meet their needs, address specific barriers, focussing not only on individual level behaviour change but also on change at institutional levels. Thus, creating the opportunity and motivation to achieve and maintain good oral health in this population.

In general, evidence suggests that behavioural support interventions are effective in improving oral health-related behaviours (14). However, interventions to improve oral health in people with SMI are influenced by many factors. A theoretical examination of the factors that impede and enhance these interventions is necessary for developing future intervention to improve oral health for this population. Theoretical Domains Framework (TDF), an integrative framework that synthesises over eighty constructs across 33 psychological theories in order to understand influences on behaviour more broadly, was developed and validated by Michie and colleagues (15, 16). The TDF has been successfully applied in many settings to identify influences on a variety of behaviours (17). The TDF is a refined version of the Capability Opportunity Motivation-Behaviour (COM-B) model, an evidence-based model based on three key sources: capability, opportunity, and motivation that influence behaviour. The COM-B model can be linked to a practical intervention design tool called the Behaviour Change Wheel framework (BCW) (18) to guide researchers in the selection of theory, intervention functions, policy categories, and behaviour change techniques (BCTs) for intervention design and delivery. As a result, the TDF is one of few frameworks linked to a comprehensive method for intervention design. BCTs can be numbered based on the Theory and Technique Tool and mapped with the potential mechanism of action of the key intervention components in the intervention package and mapped on to different theory of behaviour change model (19). This study used the Theoretical TDF to map components of the interventions targetted to improve oral health in people with SMI.

As no oral health interventions so far showed any clinically meaningful long-term effectiveness to improve oral health in this population, effective interventions and services need to be developed or adapted by understanding the context, mechanisms and population-specific barriers and facilitators. In developing an effective oral health intervention, it is vital to identify the active ingredients or BCTs used in oral health interventions tested in this population and the contextual features and barriers and facilitators of these interventions. To our knowledge, no studies have explored this and examined barriers and facilitators of different interventions targetted to improve dental health among people with SMI considering the perspectives of both mental health care service users and different service providers. Our aim was to understand the contextual factors, behaviour change techniques of the available oral health interventions and explore the barriers to and facilitators for engagement with these interventions from the perspectives of people with SMI (service users) and related service providers.

### Objectives

1. To understand and map the contextual factors and BCTs underpinning existing oral health interventions for people with SMI.
2. To explore barriers and facilitators to engagement with oral health interventions from the perspective of people with SMI, their carers and health professionals.
3. Map the barriers and facilitators to TDF and COM-B model in order to identify the capability, opportunity and motivation drivers for oral health, at the individual, inter-personal and systems level.

## Materials and methods

Ethical approval was granted by University of York Health Sciences Research and Governance Committee.

### Addressing the first study objective

Firstly, to understand the contextual factors of the existing oral health interventions for people with SMI of the 12 studies identified from the systematic review by Macnamara et al. (11), intervention details such as study setting, population, intervention timing, frequency and duration were extracted using the template for intervention description and replication (TIDieR) (20). TIDieR is helpful to systematically collect information on interventions. This was done individually by two reviewers (AM and MPM).

Secondly, to map the behaviour change techniques (BCTs) underpinning existing oral health interventions, two coders (MPM and MRF) coded the intervention descriptions for their individual BCTs using the behaviour change technique taxonomy version 1 (BCTT v1) (19).

### Addressing the second and third study objective

The intervention details identified were used to develop the video vignettes which were used at the next stage of the study to conduct in-depth interview with the service users and service providers. Prior to participating in the study, written informed consent was obtained from all the participants.

### Participants and setting

The study employed a convenience sampling technique to recruit participants. Inclusion criteria for the study was as follows for the service users recruited: people aged over 18 years, living in the UK and with a self-reported diagnosis of SMI such as schizophrenia, schizoaffective disorder or bipolar disorder, who were currently in a stable condition and had the ability to provide informed consent. Health professionals and informal carers with experience of providing health services to people with SMI were recruited as service providers. Participant recruitment took place through “Involvement@York” which is the patient and public involvement network and resource co-ordinated by the University of York (21). Participants were also recruited through social media posts and use of current contacts to spread the word about the study. Eligible participants and those who expressed interest to participate were invited by email. The invitation email contained participant information pack and consent form, and an introductory video of the project (22) which was created to provide an overview of the research to aid with recruitment. Once signed consent forms were received, a convenient date and time was scheduled for the interviews.

### Data collection

For practical reasons of time and participant availability, the format of the in-depth interviews was based on one-to-one or dyadic interviewing style which involves interviewing two participants simultaneously. Similar to one-to-one interviews, the dyadic interviews provide an opportunity to collect more individual data from each of the participants, which is not always possible with focus groups (23). Keeping in line with COVID related social distancing measures, all interviews were conducted remotely via Zoom online meeting platform (Zoom.us) (24). The interviews were co-facilitated by MRF and MPM.

The interviews were structured using bespoke video vignettes (*n* = 4) that were professionally created (25). The data related to the intervention were extracted using the TIDieR checklist and the individual BCTs that were coded as intervention components, were used to develop the four video vignettes. Each scenario showed a different setting such as: early intervention in psychosis (EIP) setting, in-patient, community out-patient setting and an integrated care model with mental and dental health services under one roof based on four studies to capture different settings and interventions (26–29). These videos were used to present the various intervention scenarios and the intervention components (use of dental checklist, provision of oral health education, brushing demonstration and practice, motivational interviewing, use of different reminders for brushing, involvement of carers, etc.) used in previous studies to improve oral health in people with SMI. The total duration of the video was seven minutes and after showing each video vignette to the participant, their views were explored in terms of acceptability, practicality and effectiveness of the intervention techniques used.

A Zoom meeting link was emailed to the participants two days prior to the meeting along with a reminder to attend the meeting. Participants’ consent was once again sought prior to initiating the recording of the zoom interviews. All participants were offered a £20 Amazon e-voucher as a token of appreciation for their time. Interviewers wrote down their reflections immediately after the interviews. The video files were deleted upon completion of each interview. The audio recordings transcribed verbatim, and transcripts pseudonymised along with removal of any identifying information. In addition, we conducted 11 one-to-one stakeholder consultations with a diverse range of stakeholders to discuss the intervention specific emerging themes on barriers and facilitators, data synthesis plan and future recommendations in order to obtain validation of our study findings. The details of the stakeholders and zoom interviews could be found elsewhere (30).

### Data analysis

To address the second study objective, the interview data were analysed to identify the barriers and facilitators for each video vignette of oral health intervention scenario, from the perspective of service users and service providers. Two reviewers (MRF and MPM) read the transcripts and discussed them along with their individual reflections and coded individually and discussed further to ensure clarity and agreement. Once initial codes were agreed on, they were then collated to form categories and sub-themes. Drawing on Braun and Clarke reflections on the use of thematic analysis in health research (31), themes were created by compiling the sub-themes for both service users and the service providers to identify each scenario specific barriers and facilitators.

To address the third study objective, the interview data were analysed based on framework qualitative analysis guided by the TDF and the COM-B model of behaviour change to identify the barriers and facilitators for engagement by people with SMI with various intervention components through the perspective of both service users and the service providers.

Framework analysis developed by Ritchie and Spencer and is a method that allows summarisation of the qualitative data through the use of a coding matrix (32) to produce structured outputs (33). In the first step of familiarisation, as mentioned above the transcripts were read by two reviewers (MRF and MPM) followed by a discussion about their individual reflections. In the second step of identifying a thematic framework, the two reviewers individually performed the coding of the transcripts which was then discussed to ensure clarity and agreement. In the third and fourth steps, once initial codes were agreed on, they were then applied to the subsequent transcripts in the process called indexing. This was followed by charting data according to the framework matrix in order to summarise the data whilst maintaining the essence of what was said during the interviews. The fifth and final step involved interpretation of data and presentation of the findings. NVivo version 12 Pro was used for analysing the data (34).

Rigour for the qualitative study process was supported through interviewers’ recording their reflections during or after the interviews, having regular discussions during the data analysis process and through constant comparison between the accounts of the participants to reduce analysis bias (35).

## Results

The TIDieR checklist was applied for the 12 intervention studies identified in the systematic review by Macnamara et al. (11) to extract intervention details (26–29, 36–43) (Supplementary Files). While most of the studies reported the setting and the core intervention, the information on tailoring (if the intervention was planned to be personalised, or adapted), modification, intervention adherence or fidelity elements were not sufficiently reported in most of the studies.

Several BCTs were identified from the coding of intervention descriptions (Table 1). The commonly used BCTs included were related to use of dental health education (*n* = 9); toothbrushing demonstrations (*n* = 6), practice (*n* = 3), and monitoring (*n* = 5); provision of toothbrushes (manual and powered) and/or toothpastes (*n* = 4); text/call reminders (*n* = 3); maintaining a toothbrushing log such as through the use of printed calendar or sticky notes (*n* = 2).

**TABLE 1:** Type and frequency of behaviour change techniques used in oral health interventions for people with SMI.

Behaviour change technique	Frequency of use in interventions	Category
1.4 Action Planning	1	1. Goals and Planning
2.1 Monitoring of behaviour by others without feedback 2.2 Feedback on behaviour 2.3 Self-monitoring of behaviour	2 3 2	2. Feedback and monitoring
3.1 Social support (unspecified)	1	3. Social support
4.1 Instruction on how to perform the behaviour	9	4. Shaping knowledge
5.1 Information about health consequences 5.3 Information about social and environmental consequences	5 1	5. Natural consequences
6.1 Demonstration of behaviour	6	6. Comparison of behaviour
7.1 Prompts/cues	3	7. Associations
8.1 Behavioural practice/rehearsal	3	8. Repetition and substitution
9.1 Credible source	3	9. Comparison of outcomes
10.1 Material incentive (behaviour) 10.2 Material reward (behaviour) 10.4 Social reward	1 2 1	10. Reward and threat
12.5 Adding objects to the environment	5	12. Antecedents

In total 17 dyadic and one-to-one interviews were conducted between July and September 2021. participant details are reported in Table 2. Interviews lasted 120 min on average and allowed for in-depth exploration of the views of both the service users and the service providers regarding each scenario of the video and barriers and facilitators related to engaging with the intervention considering acceptability, practicality, and effectiveness. The barriers and facilitators identified for each of the intervention scenario are reported in Table 3. Interventions that include involvement of carers, integrated care between mental and dental services, short and supportive oral hygiene demonstrations and provision of a toothbrush were considered as facilitators to engaging with the interventions by the service users interviewed. The preference of frequency and format of a reminder system and reinforcement via phone call or text message varied in different participants. Incorporation of a dental checklist and signposting to a dentist alone was not considered sufficient unless further support is provided in accessing to dental care are following completion of the checklist. Opportunity for appropriate training and opportunity of provision of integrated care system for collaborative work of both mental and dental health care staff was considered as facilitator to engage by the service providers. However, increased workload was considered as a barrier by service providers.

**TABLE 2:** Demographics of the study participants.

ID	Participant group	Age (years)	Gender	Diagnosis/Profession
1	Service user	31–40	M	Schizophrenia
2	Service user	>60	F	Schizophrenia
3	Service user	31–40	F	Schizophrenia
4	Service user	41–50	M	Schizophrenia
5	Service user	41–50	M	Bipolar disorder
6	Service user	>60	F	Bipolar disorder
7	Service user	41–50	F	Bipolar disorder
8	Health professional	31–40	F	Community service dentist
9	Health professional	31–40	F	High street dentist
10	Health professional	31–40	F	Dental hygienist
11	Health professional	31–40	M	Special care dentist
12	Carer	51–60	F	Caring for person with schizophrenia
13	Health professional	31–40	M	Occupational therapist
14	Health professional	31–40	M	Clinical psychologist
15	Health professional	31–40	F	Mental health nurse
16	Health professional	41–50	F	Mental health nurse
17	Health professional	31–40	M	Special care dentist

M, male; F, female.

**TABLE 3:** Intervention Scenario specific barriers and facilitators.

Intervention scenarios	Barriers/Facilitators	Service users perspective	Service providers perspective
1. Early Intervention in Psychosis (EIP) setting: use of dental checklist by care coordinators (mostly mental health nurses) to monitor oral health status and behaviours, sign posting to dentist	Barriers	1. Need for practical help with finding/setting up an appointment with the dentist 2. Lack of availability of trauma informed dentists 3. Long follow up time of 12 months	1. Need for training around oral health 2. Increased workloads of the mental health nurses
	Facilitators	Involvement of the care coordinator	Use of checklist fits well with their current work routine
2. Community mental health (in-patient) setting: Oral hygienist delivering oral hygiene instructions to patients and training to mental health nurses	Barriers	1. In acute crisis stage of an inpatient setting just getting through the day is the top priority not oral health 2. Trust issue with someone new coming in to talk about oral health. 3. Lack of support after being discharged 4. Stable mental health does not necessarily mean being fit to visit a dentist unsupported. 5. Lack of coordination between mental, physical and oral health services- (who takes the responsibility for the person?)	Need for training around mental health and side effects of psychotropic medication
	Facilitators	Provision of toothbrush and toothpaste	Short training session (20 mins)
3. Community mental health (out-patient) setting Dental hygienist provides dental education, patient is given sticky notes as reminder system and also receives weekly calls for 4 weeks for positive reinforcement	Barriers	1. Long oral health education sessions are difficult to follow. 2. Costs of maintaining electric toothbrush. 3. Lack of involvement of any carer 4. Post it notes can trigger obsessive behaviours 5. Weekly call seems intrusive	Need for training around mental health
	Facilitators	1. Use of post-it notes simple than keeping a diary/log. 2. Electric toothbrush can make cleaning easier 3. Good follow up	Weekly phone calls can be managed
4. Integrated care model (with mental and dental health services under one roof) setting: involvement of carer during provision of dental education	Barriers		1. Lack of availability of such integrated care model in UK 2. Funding/commissioning issues
	Facilitators	1. Good model of getting services easily. 2. Involvement of carer 3. Use of video demonstrations 4. Short follow up	

**FIGURE 1:**
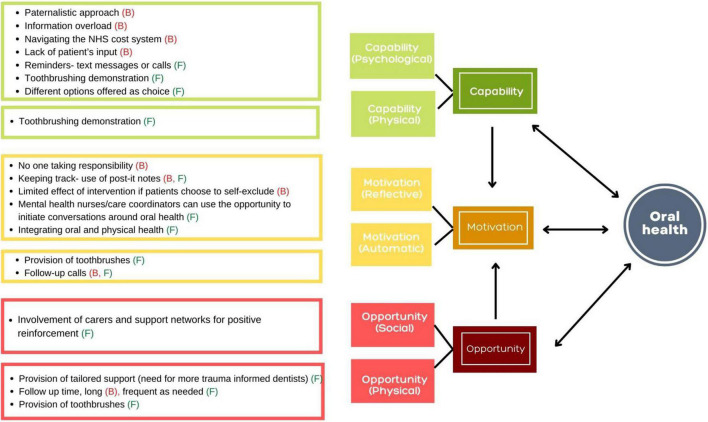
The barriers (B) and facilitators (F) reported by the service users on three main themes according to the COM-B model.

**FIGURE 2:**
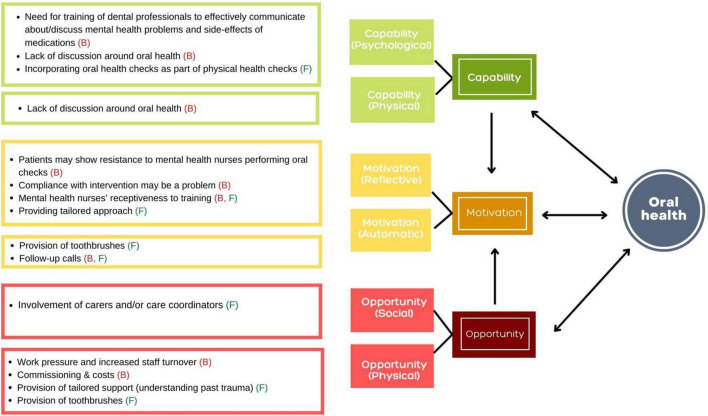
The barriers (B) and facilitators (F) reported by the service providers on three main themes according to the COM-B model.

To address the third study objective, the grouping of the barriers and facilitators were reported under the TDF domains. Further grouping of the barriers and facilitators under the TDF domains according to the COM-B model provided an insight into the capability, opportunity and motivation drivers of behaviour change that can be tapped into to enable development of interventions that provide integrated support to people with SMI. The barriers (B) and facilitators (F) reported by the service users and service providers on three main themes according to the COM-B model are presented in Figures 1, 2, respectively. An overview of the three main themes according to the COM-B model, sub-themes according to the TDF and barriers (B) and facilitators (F) is provided in Table 4 and significant findings are reported below.

**TABLE 4:** Barriers and facilitators under the TDF domains and mapped to the COM-B model.

COM-B component	Theoretical domains framework	Barriers and facilitators (identified through the interviews)	
		Service users	Service providers
**Capability** (Psychological)	Knowledge Skills Memory, attention and decision processes Behavioural regulation	Paternalistic approach often used (B) Toothbrushing demonstration (F) Information overload (B) Navigating the NHS cost system (B) Reminders- text messages or calls (F) Lack of patient’s input (B) Different options offered as choice (F)	Need for training of dental professionals to effectively communicate about/discuss mental health problems and side-effects of medications (B) Lack of discussion around oral health (B) Incorporating oral health checks as part of physical health checks (F)
**Capability** (Physical)	Skills	Toothbrushing demonstration (F)	Lack of discussion around oral health (B)
**Opportunity** (Social)	Social Influences	Involvement of carers and support networks for positive reinforcement (F)	Involvement of carers and/or care coordinators (F)
**Opportunity** (Physical)	Environmental context and resources	Provision of tailored support (need for more trauma informed dentists (F) Follow up time, long (B), frequent (F) Provision of toothbrushes (F)	Provision of tailored support (understanding past trauma) (F) Increased staff turnover (B) Commissioning & costs (B) Provision of toothbrushes (F)
**Motivation** (Reflective)	Social and Professional role & identity Beliefs about capabilities Beliefs about consequences	Mental health nurses/care coordinators can use the opportunity to initiate conversations around oral health (F) Integrating oral and physical health (F) No one taking responsibility (B) Keeping track- use of post-it notes (B, F) If patients choose to self-exclude themselves, intervention will not have much affect (B)	Mental health nurses’ receptiveness to training (B, F) Patients may show resistance to mental health nurses performing oral checks (B) Providing tailored approach (F) Compliance with intervention may be a problem (B)
**Motivation** (Automatic)	Reinforcement	Provision of toothbrushes (F) Follow-up calls (B, F)	Provision of toothbrushes (F) Follow-up calls (B, F)

B, barriers; F, facilitators.

### Theme 1: Capability

#### Sub-theme: Knowledge and skills

The service users felt that oral health education was an important aspect of an intervention to increase their knowledge and toothbrushing demonstrations were helpful to provide them with necessary skills. However, how the narrative was framed was important. A ‘paternalistic’ approach was reported as a significant barrier.

*“it’s you know it’s infantilizing what’s going on there now. As a reframing of that it could be that actually maybe we need to go in at that sort of level to establish effective new ways of you know it’s when we’re facilitating the change…. it depends who’s watching yeah and then how they approach it, you know it’s not about ooh that’s right, that’s wrong, its all about using motivational interviewing skills.”* (Service user- J-04 with diagnosis of bipolar disorder).

Lack of training of staff involved in provision of care to people with SMI was identified as a barrier. Both the dental and mental service providers agreed that there is a need for training staff in ways that they communicate with their patients regarding oral health and mental illness. These include training in softer communication skills and also providing more information around discussing possible side effects of anti-psychotic medications on their oral health.

“*I would say so [there is a need for training]. Just language used and what sorts of things to expect and not be surprised by them.”* (Service provider- S-08, worked as mental health nurse for 23 years).

*“For the education for the nurse is under the kind of the knowing about how psychotropic medications are going to affect oral hygiene and things like that, I think that sounded perfect because that’s certainly an area that we’re lacking in.”* (Service provider- M-09, working as a mental health nurse).

#### Sub-theme: Memory, attention and decision processes, and behaviour regulation

Although the service users expressed a desire to be more involved in provision of their care, they felt that it needed to be done in a way that did not entail overloading them with information which would otherwise be just another barrier.

“*So I think that’s all I can say with that scenario (Scenario 3) overall that too much information, and how suitable is the information to the patient? Okay, it’s a learning opportunity around the patient learning, and having the ability to learn and take their own choices and decisions around oral health hygiene, but I think it could work against the patient because too much knowledge, too much learning.”* (Service user- S-01 with diagnosis of schizophrenia).

Facilitators included provision of more practical help such as reminder texts or calls depending on the individual person’s preference and help with navigating the NHS cost system were spoken about as something in which provision of support was welcome.

“*Oh yeah be great option yeah especially text messages I don’t know about other people*…*and don’t make it a voice call you know when you leave a voice message, I never open mine. I can’t cope with the idea of somebody leaving a message on the phone.” (Service user- J-06, with diagnosis of bipolar disorder).*

Lack of discussion around oral health by the health professionals was attributed to factors such as not being trained enough to initiate conversations on the topic. The dental professionals also stated that high caseloads meant that there was only a limited time allocated for each patient and this precluded the possibility of having detailed conversations with their patients. The option of mental health nurses incorporating oral health checks through a checklist as part of their patient’s routine physical examination received a mixed response and was identified both as a barrier and a facilitator, both by the service providers and the service users. The views expressed were that although it has potential to work because mental health service users already have trusting relationship with their care coordinators, the whole idea should not be limited to ‘just another piece of paperwork’ but taken forward as an opportunity to initiate conversations around oral health.

“*Opening up a conversation about teeth and oral health and there is some records and then there’s obviously, a point where you can then revisit it so you know that there is a benchmark that you’re laying down a year before, and it will obviously, depending on how much details within, it will give you some idea of what the population need is, and I suppose an understanding as to how many of those patients do regularly see a dentist because some of them will have dentists, and some of them wont*…. *I suppose it will vary, and that will give you a lot of interesting information but I think you’re in respects to it’s opening up a conversation and giving somebody to support and in regards to trying to find a dentist and but I’m just not sure whether it goes far enough*.” (Service provider- C-01 working as special care dentist).

### Theme 2: Opportunity

#### Sub-theme: Social influences

Both the service users and the service providers were of the opinion that the involvement of carers such as informal carers (family and friends) or formal carers such as care coordinators or mental health nurses was a pivotal point in the intervention scenarios. Carers can help provide people with SMI the motivation to engage in healthy oral health behaviours by providing practical support such as helping them brush their teeth, accompanying them to visit the dentist, etc.

*“I’d like to see the emphasis on the importance of having somebody with them all the way through the videos (scenario 4) not you know sat in front of that professional with a flip chart you know, in the hospital with the loved one beside them.*…*you know you will learn the carer will learn something about the loved one they didn’t and vice versa, and it’s a bonding experience. But also, it can be that can be equal benefit to a professional that supports you as well, they might get to learn a bit more about you know it’s that togetherness stronger together, you know, rather than you.”* (Service user-K-05 with diagnosis of Schizophrenia).

“*Yeah I think the it definitely varies too, I think, for some people that’s enough [signposting to find a dentist], some people already probably attending the dentist or the people would need to be probably with the care coordinators to call up on their behalf, and to provide some kind of active encouragement for them to go and some of our best care coordinators, the ones who would maybe push a bit hard, there’d be quite encouraging for people to go to that first appointment and helping them kind of overcome their fears.” (Service provider- C-05 working as clinical psychologist).*

#### Sub-theme: Environmental context and resources

Regarding interventions related to visiting the dentist, lack of tailored support was highlighted as a major barrier both by the service users and the service providers. Utilising this opportunity to provide tailored support to people with SMI such as availability of trauma informed dentists and shorter follow ups can go a long way to support this population in their oral health needs.

*“For me, trauma is another barrier so had really bad experiences as a kid with dentist or dentistry and then as an adult more recently about seven years ago, I had a really bad experience with a dentist he wasn’t trauma informed.”* (Service user-K-05 with diagnosis of schizophrenia).

“*Yeah, I mean for dental we normally use NICE guidance, but what we found is you know a number of patients with mental problems that normally require quite, most of them, not all of them require more frequent follow up just because depending on the condition and their oral health condition they may need kind of more frequent follow-up. But I think once they’re seen by a dental practitioner, then that can be discussed with the care coordinator, and then they can kind of maybe agree on a time scale for review.” (Service provider- H-04 working as special care dentist).*

The service providers also spoke about the system level barriers such as the way dental commissioning works, dental treatment costs, and staff turnovers that can prove to be a barrier in provision of an adequate level of care to people with SMI. For example, the need to meet a certain threshold of severity of mental illness before being referred to community dental services which are better equipped to deal with their dental treatment needs, and thus having to utilise the services of a high street dentist. However, consecutive missed appointments due to mental ill health can leads to being removed from a dental surgery’s patient list.

*“I often feel, they are left in limbo in these cases, so like the actual care pathway doesn’t take into account their needs*… *I would like to see them every day when they want to come in, but obviously we have to you know comply with any NHS rules and it’s like if you don’t attend twice you are normally struck off from the treatment pathway, and you have to start from scratch. Often they’re de-registered from the practice and then these days it’s really hard to find a dentist like recently, one of my patients were struck off from that list because she didn’t attend three one hour appointments with me, so the practice deregistered her, and I know that she’s suffered from mental health so we can’t see her anymore so sometimes it’s a bit of beaurocratic decisions as well that come in the way between dentist and the patient, I guess, in these cases.” (Service provider- E-02 working as a high street dentist).*

Provision of toothbrushes whether manual or powered depending on patient’s preference was identified as a facilitator by both the service users and the service providers, with participants believing that a new toothbrush helped increase motivation to engage in regular toothbrushing behaviours.

“*yeah I like that element [provision of toothbrushes] you know, again, the link between people suffering and poverty is probably something that not many people have access to and just the notion of being given something valuable, I think, can make people feel valued and again that notion that like someone cares about you and your team yeah and the novelty of having it. I think that people would use it and so yeah I definitely support that*. (Service user- S-07 with diagnosis of schizophrenia).

### Theme 3: Motivation

#### Sub-theme: Social and professional role and identity

Service users felt that care-coordinators were a good resource to initiate conversations around oral health as they have established rapport and a trusting relationship with them. In particular they discussed the provision of more practical support such as finding a dentist, setting up an appointment etc. rather than just ticking boxes on a checklist.

*“I guess it would just be that alongside the checklist if in conversation that the patient and care coordinator had concerns I say it was flagged that they weren’t registered with a dentist and didn’t feel able to do that, that wasn’t the end of the conversation that there was like the capacity to follow up an office support for that and like literally the big thing that made the difference for me was just someone else making that first phone call and contacting the dentist and then it all sort of went from there, which isn’t a massive things I wouldn’t be overly concerned that it was going to be hugely time consuming, but I just think it’s just the checklist on its own there’s a chance that that can increase the sense of shame, but not actually lead to anything being done differently*.” (Service user-Sa-07 with diagnosis of Schizophrenia).

Although health professionals agreed with this idea of involving mental health nurses in provision of oral health support, some shared their concerns regarding the barriers related to capacity and willingness of mental health nurses to be involved in provision of oral health support in the face heavy caseloads and with most of the services stretched to the limit due to high staff turnovers. Furthermore, the service providers also felt that another barrier could be the possibility of service users not willing or feeling comfortable for non-dental personnel to perform oral health checks (such as taking a quick look in the mouth to identify any problems) even if they are trained to do so.

*“I think, where the difficulty is the high turnover of staff. Sometimes capturing on staff to do that and honestly trying to ensure that that’s kind of carried out by the staff, because I think there’s other priorities with the patient. They have to look at it as something that can be done within their kind of shift, with all the other kind of requirement for them to do as well yeah and I think it’d be good if implemented.”* (Service provider-H-04 working as special care dentist).

Nevertheless, intervention related to an integrated model of care where health professionals who are involved in a patient’s physical (including oral health) and those involved in their mental health work together to provide integrated care was spoken about as a way forward to ensure oral health moves from ‘nobody’s business’ to ‘everyone’s business’.

*“You know if that consistent support and making every contact count does that make sense, and also that it’s if you make it one person’s business and responsibility quite quickly becomes nobody’s business or responsibility.”* (Service user-K-05 with diagnosis of Schizophrenia).

#### Sub-theme: Beliefs about capabilities and beliefs about consequences

The intervention elements that required keeping a record of daily toothbrushing elicited a mixed response from service users. Techniques such as use of toothbrushing activity calendars or pulling out a sticky note every time an individual brushed their teeth were felt by some to have the tendency of provoking their obsessive compulsiveness. On the other hand, other service users felt that marking on a calendar or pulling out a sticky note was better way of keeping a track and needed little input compared to maintaining a diary.

*“I would really like it yeah because, to be honest, the other thing about it is they give us so many things that you have to tick a box with and it just you know, in the end you just get fed up so it’s something different, something new and something that you don’t actually have to do anything about apart from pull and plop do you know what I mean yeah no I think that’s really good.”* (Service user-J-06 with diagnosis of bipolar disorder).

Furthermore, it was highlighted that even if the problem could be solved by providing the service users a choice over the method of record keeping, a person’s non-compliance or lack of engagement with the techniques can still prove to be barrier.

*“One thing is like you’re probably having to think about two areas of compliance and cooperation, so one of them is the actual brushing but then one of them is putting the sticky note in the box*…*but possibly if the count is lower, I’ll be careful about understanding is the count because they didn’t brush their teeth or is the count because they forgot they brushed their teeth, or they forgot to put the slip in so that’s probably one of the sensitivities to be aware of*.” (Service provider- B-10 working as special care dentist).

#### Sub-theme: Reinforcement

Provision of follow-up calls or texts were discussed as a facilitator for providing positive reinforcement to the service users to encourage them to maintain their oral hygiene. The overall consensus was to offer this support as a choice to people with SMI as the willingness to engage may vary depending on the person and on the day for example on a bad day it could prove to be an overwhelming task.

“*I think it’s one of these things that all sizes don’t fit all isn’t it, you know people are going to react differently, to telephone interventions, some are going to see it as really helpful and keeping in touch with somebody from the outside world but the more introverted patient might not welcome it quite as much*.” (Service user- M-02 with diagnosis of schizophrenia).

While discussing the initial findings and recommendations of the study with the stakeholders, they agreed with the overall study findings and recommendations. However, stakeholders’ consultation revealed that due to lack of system level integration between mental and dental healthcare services and work load of the related healthcare staff and with the current commissioning system, it might be challenging to incorporate some of the interventions. Careful consideration is needed to develop a system level intervention and evaluation of the effectiveness and cost-effectiveness and sustainability of the intervention.

## Discussion

The study aimed to explore the barriers and facilitators of oral health interventions and their components to identify the capability, motivation and opportunity drivers of behaviour change.

This study reports on analysis of contextual factors, BCTs and mechanisms used in previously tested interventions for improvement of oral health in people with SMI. There were 16 individual BCTs identified from the description of previously tested interventions for oral health improvement in people with SMI. The most commonly used BCTs were “*4.1 instructions on how to perform the behaviour”* and “*6.1 demonstration of behaviour”* pertaining to toothbrushing practice. It was noticeable that social support in terms of “*3.1 social support (unspecified)”* and “*3.2 social support (practical)”* were not some of the frequently used BCTs. Though dental education and oral hygiene instructions along with provision of toothbrush showed some short-term effectiveness and seemed to be viable in psychiatric outpatient settings (28), given the barriers that people with SMI face for finding a dentist, setting up an appointment and attending one, there needs to be provision of more tailored support in terms of their oral and dental care.

The study also reports a qualitative exploration of barriers and facilitators for engagement with oral health interventions through the perspectives of people with SMI (service users), carers, and the service providers. In the current study, an integrated care model that was tested by Agarwal and colleagues was identified as the best kind of service that can be offered to people with SMI for their oral and physical health needs (29). This indicated the need to consider oral health very much a part of the general health and wellbeing. Furthermore, involvement of service users in their care planning in conjunction with their carers, be it formal carers (such as care coordinators) or informal carers (family members) can have a positive influence in improving patient compliance through provision of tailored services and reinforcement of messages.

Training of mental health care nurses around oral health was identified as a facilitator in this study. From the findings of a qualitative study exploring the views of mental health nurses in Australia regarding dental access and dental ill health of people with SMI (44), it can be deduced that given the salience of concern nurses have for the dental health of people with SMI, mental health nurses may offer an important route for service users to access dental health care through providing advice and facilitating referrals. Adams et al. in their study on monitoring oral health in people with SMI in the UK reported that while a simple nurse led checklist to monitor oral health did not demonstrate any improvement (26), it did help highlight the oral health needs of people with SMI. Although mental health nurses who are closest to the patients in provision of care, can help initiate conversations related to oral health, high staff turnover and huge workload is a barrier that needs to be considered in planning of related oral health interventions. Almomani et al. discusses the need for special training for dentist and dental hygienists in working with people with SMI along with improving the knowledge of oral care in nursing staff (28). Furthermore, De May et al. concluded that ‘oral health of SMI patients can improve significantly with basic oral health interventions carried out by collaborating oral hygienists and mental health nurses’ (27).

The strengths of this study include the fact that a systematic approach was used to extract intervention study related information using the standardised checklists such as TIDieR checklist and BCTTv1. The use of theoretical frameworks such as TDF and COM-B model allowed the exploration of the barriers and facilitators at personal/individual level, interpersonal/service user-service provider level and environmental/systems level and based on these, explore the capability, opportunity and motivational drivers for promoting oral health in people with SMI.

Use of video vignettes enhanced the participant’s understanding of different intervention scenarios and is a unique way of engaging participants in the thought and discussion process especially when conducting research from a distance. Furthermore, consultations with a diverse range of stakeholders helped to obtain validation of our study findings and recommendations. The study has some limitations that warrant mentioning. The sample size was small and was based on the number of people with SMI who agreed to participate within the data collection period. Secondly, incorporation of a wider range of ethnic groups could have provided additional insights. However, within the constraints of time and resources, we conducted a good quality but modestly sized study and tried to bring to light of this often-neglected area of improving oral health in people with SMI.

To our knowledge, this is the first study to explore the barriers and facilitators for engagement with oral health interventions, from the perspectives of people with SMI, their carers and different professionals involved in their care. We included mental health care service users, male and female from different age groups, with different diagnoses of severe mental illness. Assessing service users’ and providers’ perceptions of barriers and facilitators to oral health interventions will help to design future interventions to improve oral health in people with SMI. The implications of the study go beyond oral health and the context of the UK setting due to the applicability of recommendations to other health promotion delivery across the world (exercise, diet, smoking cessation etc.). Paternalistic ways of offering advice and information overload can be a barrier to acceptability in any health promotion activity.

### Recommendations

More understanding and awareness of trauma-informed dental practice is needed for this vulnerable group. Employing standard principles of Sensitive Practice from the framework of The Umbrella of Safety would facilitate feelings of safety for the service users (45). Furthermore, future interventions, in addition to provision of oral hygiene instructions, should focus on the provision of more practical support in terms of accessing dental services, such as support with finding and attending a dentist.

The British Society of Disability and Oral Health guidelines published in 2000 made a number of recommendations for oral health care for people with mental health problems, including providing oral health advice, support, promotion and education addressing the oral health needs of clients (46). Providing advice and education (diet advice for reducing frequency of sugar intake and tooth brushing advice on the correct techniques and duration of brushing) are not sufficient to ensure improvement in oral health. It is imperative that compliance and stability in oral health following education and advice is monitored and reinforced as necessary until stabilisation is achieved (47). Therefore, future work should focus on co-producing an intervention (48) to improve oral health in people with SMI and related training materials as it increases the relevance of research by ensuring that it reflects the needs, values and interests of patients and improves the quality of research through broadening the range of expert input.

People with SMI are not a homogenous group (49, 50) and face unique barriers to maintaining good oral health (30, 51) that the interventions rarely considered. Therefore, a patient-centred approach to oral health promotion (46) should be followed as use of person-centred care in mental health treatment models has promising outcomes for engagement (52).

Although an integrated care model at the study setting (29) was mostly preferred service by the service users as identified in this study, integration between dental, mental health, and other health services is currently lacking. Therefore, an important element of any future guidance should consider how dental care can be integrated in mental health care services (53, 54). The recommendations could be applicable to other health concerns in this population as the integration of physical (including dental) health care with mental health care and support of mental health workers and care coordinators would be beneficial for people with SMI. We acknowledge that the context and specifically the health care service delivery system varies across different countries. However, considering the health inequalities of people with SMI both in developing and developed countries, an integrated model approach would be beneficial. The model whereby the dental service sits under the same roof of mental health care (29) was identified by service users as the optimum care offered to people with SMI for their oral health needs. Health professionals who are involved in mental health, oral health, and physical health care need to work together with good communication channels between various services and with the service user to optimise their health care including oral health.

## Conclusion

The findings of the present study suggest that the oral health needs of people with SMI continue to be unmet. Interventions that additionally focus on inter-personal and system levels barriers have the potential to be beneficial for improving the oral health of this vulnerable group of people. The results of this study will inform the development of a fit-for-purpose system level oral health intervention to improve oral health of people with SMI.

## Data availability statement

The original contributions presented in this study are included in the article/Supplementary material, further inquiries can be directed to the corresponding author.

## Ethics statement

This study was conducted according to the guidelines of the Declaration of Helsinki and approved by the Health Sciences Research Governance Committee of the University of York (protocol code: HSRGC/2021/438/C and date of approval: 19 March 2021). The patients/participants provided their written informed consent to participate in this study. Written informed consent was obtained from the individual(s) for the publication of any potentially identifiable images or data included in this article.

## Author contributions

MM and LG: conceptualisation and supervision. MM, MF, LN, and LG: methodology. MM and MF: investigation, data curation, formal analysis, visualisation, writing—original draft preparation, and project administration. SG, LG, EP, and MM: resources and funding acquisition. All authors: writing—review and editing.
