# Supplementary material for: Exploring the contextual factors, behaviour change techniques, barriers and facilitators of interventions to improve oral health in people with severe mental illness: A qualitative study
Source: Front Psychiatry. 2022 Oct 11;13:971328. doi: 10.3389/fpsyt.2022.971328 (PMC9592713; doi:10.3389/fpsyt.2022.971328)
Supplement: Supplementary file 1 [file Table_1.DOCX]

**
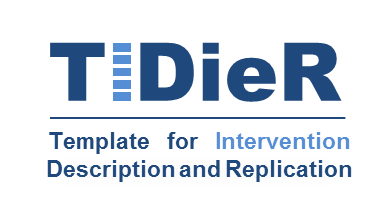
The TIDieR (Template for Intervention Description and Replication) Checklist*:**

Information to include when describing an intervention and the location of the information

| **Item number** | **Item: Adams 2018** | **Where located **** | |
| --- | --- | --- | --- |
|  |  | Primary paper  (page or appendix  number) | Other ^†^ (details) |
|  | **BRIEF NAME** | 107 |  |
| **1.** | Provide the name or a phrase that describes the intervention.  "*Checklist for monitoring oral health*" | ________ | ______________ |
|  | **WHY** | 107 |  |
| **2.** | Describe any rationale, theory, or goal of the elements essential to the intervention.  *“It is imperative that compliance and stability in oral health following education and advice is monitored and reinforced as necessary until stabilisation is achieved. A Cochrane review investigating the effects of such approaches found no relevant randomised trials comparing an oral health advice or monitoring intervention with standard care for people with serious mental illness. It was hoped that such monitoring, partly designed to precipitate help and advice where needed, may have practical measurable benefits. It is equally possible that such activity has no discernible effect.”* | __________ | _____________ |
|  | **WHAT** | 109 (checklist) | Supplementary materials of published protocol (training manual and advice sheet) |
| **3.** | Materials: Describe any physical or informational materials used in the intervention, including those provided to participants or used in intervention delivery or in training of intervention providers. Provide information on where the materials can be accessed (e.g. online appendix, URL). | ___________  108 | _____________ |
| *4.* | *Procedures: Describe each of the procedures, activities, and/or processes used in the intervention, including any enabling or support activities.*  *“After randomisation, EIP teams allocated to receive the dental intervention were approached by the trial team to arrange the dental awareness training. Information sheets were given out to Care Co-ordinators and additional consent forms signed. This fitted within the usual multidisciplinary team meetings but took around 30 min. The training briefly covered the agreement of the importance of oral health care in this group, encouragement of awareness of this aspect of care, aims and background of the trial, how to complete the checklist, service user ID number allocation, how to return completed checklists to the trial team and discussion about what to do in certain situations regarding adverse events. The dental checklist was adapted from the British Society for Disability and Oral Health (BSDH) guidelines and carried, embedded within it, the Clinical Global Impression categorical checklist. The Care Co-ordinators were encouraged to use the checklist for all their service users at their earliest convenience. These experienced clinicians – mostly nurses*  *– were given no additional training beyond the initial awareness-raising meeting but this simple approach is in keeping with the premise for using the CGI”.* | ____________ | _____________ |
|  | **WHO PROVIDED** |  |  |
| **5.** | For each category of intervention provider (e.g. psychologist, nursing assistant), describe their expertise, background and any specific training given.  *“…dental awareness training … took around 30 min. The training briefly covered the agreement of the importance of oral health care in this group, encouragement of awareness of this aspect of care, aims and background of the trial, how to complete the checklist, service user ID number allocation, how to return completed checklists to the trial team and discussion about what to do in certain situations regarding adverse events.” Delivered to care co-ordinators: “experienced clinicians – mostly nurses”* | 108  ___________ | _____________ |
|  | **HOW** | 108 |  |
| **6.** | Describe the modes of delivery (e.g. face-to-face or by some other mechanism, such as internet or telephone) of the intervention and whether it was provided individually or in a group.  “*The Care Co-ordinators were encouraged to use the checklist for all their service users at their earliest convenience*.” “*The teams have a Senior Manager overseeing the Care Co-ordinators (mostly nursing staff) who are the main contact person for service users throughout their involvement with the service” No information available on whether face to face or telephone”.*   Assumption that it is individual and possibly face to face, given the setting, but this is not explicitly stated. | ___________ | _____________ |
|  | **WHERE** |  |  |
| **7.** | Describe the type(s) of location(s) where the intervention occurred, including any necessary infrastructure or relevant features.  *“The trial was conducted as part of standard care provided by the Early Intervention in Psychosis (EIP) teams, first in Nottinghamshire, Derbyshire and Lincolnshire (UK), and then in other teams across northern England (Bradford, Doncaster, Durham, Leeds, Northumberland, Wakefield). The teams cover a mixture of urban and rural areas with a diverse population. The multidisciplinary EIP out- patient teams provide intensive treatment and support to people with a first experience of symptoms such as hearing voices or those who develop unusual beliefs which may indicate the onset of psychosis.”* | 107  ___________ | _____________ |
|  | **WHEN and HOW MUCH** |  |  |
| **8.** | Describe the number of times the intervention was delivered and over what period of time including the number of sessions, their schedule, and their duration, intensity or dose. “*The Care Co-ordinators were encouraged to use the checklist for all their service users at their earliest convenience*.” Checklist used once as part of intervention, and once for outcome measurement: “*The trial team prompted the intervention group Care Co-ordinators*  *for the 12 month follow up where dental checklists were to be completed again for all service users – the intervention checklist doubling as the outcome form”* | 108  ___________ | _____________ |
|  | **TAILORING** |  |  |
| **9.** | If the intervention was planned to be personalised, titrated or adapted, then describe what, why, when, and how. | N/A  ___________ | _____________ |
|  | **MODIFICATIONS** |  |  |
| **10.^ǂ^** | If the intervention was modified during the course of the study, describe the changes (what, why, when, and how). | N/A  ___________ | _____________ |
|  | **HOW WELL** |  |  |
| **11.** | Planned: If intervention adherence or fidelity was assessed, describe how and by whom, and if any strategies were used to maintain or improve fidelity, describe them. | ?  _________ | _____________ |
| **12.^ǂ^** | Actual: If intervention adherence or fidelity was assessed, describe the extent to which the intervention was delivered as planned.  *“We anticipated 600–800 dental checklists to be completed during the trial” “The intervention teams returned 882 baseline intervention forms”* . Number in agreement with planned anticipated numbers, however the total number of potential recipients is not reported. | ?  _________ | _____________ |

** **Authors** - use N/A if an item is not applicable for the intervention being described. **Reviewers** – use ‘?’ if information about the element is not reported/not sufficiently reported.

† If the information is not provided in the primary paper, give details of where this information is available. This may include locations such as a published protocol or other published papers (provide citation details) or a website (provide the URL).

ǂ If completing the TIDieR checklist for a protocol, these items are not relevant to the protocol and cannot be described until the study is complete.

* We strongly recommend using this checklist in conjunction with the TIDieR guide (see *BMJ* 2014;348:g1687) which contains an explanation and elaboration for each item.

* The focus of TIDieR is on reporting details of the intervention elements (and where relevant, comparison elements) of a study. Other elements and methodological features of studies are covered by other reporting statements and checklists and have not been duplicated as part of the TIDieR checklist. When a **randomised trial** is being reported, the TIDieR checklist should be used in conjunction with the CONSORT statement (see [www.consort-statement.org](http://www.consort-statement.org)) as an extension of **Item 5 of the CONSORT 2010 Statement.** When a **clinical trial** **protocol** is being reported, the TIDieR checklist should be used in conjunction with the SPIRIT statement as an extension of **Item 11 of the SPIRIT 2013 Statement** (see [www.spirit-statement.org](http://www.spirit-statement.org)). For alternate study designs, TIDieR can be used in conjunction with the appropriate checklist for that study design (see [www.equator-network.org](http://www.equator-network.org)).
