# Supplementary material for: Exploring the contextual factors, behaviour change techniques, barriers and facilitators of interventions to improve oral health in people with severe mental illness: A qualitative study
Source: Front Psychiatry. 2022 Oct 11;13:971328. doi: 10.3389/fpsyt.2022.971328 (PMC9592713; doi:10.3389/fpsyt.2022.971328)
Supplement: Supplementary file 2 [file Table_2.DOCX]

**
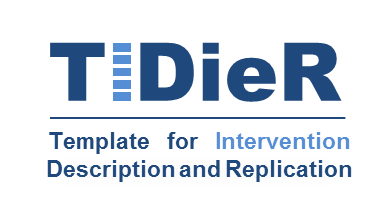
The TIDieR (Template for Intervention Description and Replication) Checklist*:**

Information to include when describing an intervention and the location of the information

| **Item number** | **Item** | **Where located **** | |
| --- | --- | --- | --- |
|  |  | Primary paper  (page or appendix  number) | Other ^†^ (details) |
|  | **BRIEF NAME** | 2 |  |
| **1.** | Provide the name or a phrase that describes the intervention.  *Oral health education (practical chair side oral health education approach which could be imparted by a dentist or auxiliaries in the dental settings)* | ____________ | ______________ |
|  | **WHY** | ? |  |
| **2.** | Describe any rationale, theory, or goal of the elements essential to the intervention. | ____________ | _____________ |
|  | **WHAT** | 2-3 |  |
| **3.** | Materials: Describe any physical or informational materials used in the intervention, including those provided to participants or used in intervention delivery or in training of intervention providers.  *1.brushing model*  *2. two videos showing technique*  *3.participants were then provided with a soft toothbrush and a fluoridated tooth paste.*  *4. A personalized handout in the form of calendar printed in the local language explicating details of oral hygiene practices, negative oral health beliefs, and pictures* *for the same was delivered.*  Provide information on where the materials can be accessed (e.g. online appendix, URL).  No reference of sources availability | ____________ | _____________ |
| **4.** | Procedures: Describe each of the procedures, activities, and/or processes used in the intervention, including any enabling or support activities.  *Oral health education was conducted in the experimental group. They were first taught the modified bass technique for brushing teeth on a brushing model followed by two videos showing modified Bass or modified Stillman technique for brushing teeth and method of tongue cleaning using tongue cleaner or back of toothbrush followed by rinsing and washing the toothbrush. The participants were then provided with a soft toothbrush and a fluoridated tooth paste and were asked to demonstrate brushing technique on their teeth in front of the examiner. They were assisted, monitored, and the process was repeated if required. They were asked to brush twice daily, while the subjects in the control group were advised standard care, i.e., to brush twice daily, mouth rinsing after every meal, and tongue cleaning. Efforts were made to bring about the change in false beliefs pertaining to dental treatment and maintenance of oral hygiene. For enhancement in the intervention group, oral health education was carried out in the presence of accompanying relative who was encouraged to accompany the participant in subsequent visits and also motivate him/her to achieve the desired oral health goals. At the second visit, i.e., at 1 month after first oral health education, the subjects were asked to perform brushing technique on the model and any flaws, if observed, were identified and corrected. A personalized handout in the form of calendar printed in the local language explicating details of oral hygiene practices, negative oral health beliefs, and pictures for the same was delivered. The idea behind the calendar was to act as reinforcement for intervention. There was no second visit for the control group.* | 2-3_____ | _____________ |
|  | **WHO PROVIDED** |  |  |
| **5.** | For each category of intervention provider (e.g. psychologist, nursing assistant), describe their expertise, background and any specific training given.  *The investigator: dentists*  *The investigator attended 8 hours’ educational session of interaction between psychiatrist, patients, and their relatives to develop feasible and effective health education strategy. A well-structured health education material was developed with PEMAT (Patient Education Materials Assessment Tool) score of 93.3% for understanding ability and 80% for action ability. Training and calibration of principal investigator was conducted in the Department of Public Health Dentistry for standardized recording of clinical scores.* | ______3____ | _____________ |
|  | **HOW** | 3 |  |
| **6.** | Describe the modes of delivery (e.g. face-to-face or by some other mechanism, such as internet or telephone) of the intervention and whether it was provided individually or in a group.  *Face to face, Individual for the patients*. | ____________ | _____________ |
|  | **WHERE** |  |  |
| **7.** | Describe the type(s) of location(s) where the intervention occurred, including any necessary infrastructure or relevant features.  Participants *were selected from the Outpatient Department of Psychiatry.*  *Location: ? (Location of intervention occurred was not clearly mentioned. From the corresponding authors details most probably the study was carried out in* Postgraduate Institute of Dental Sciences, Pt. B.D Sharma University of Health Sciences, Rohtak, Haryana, India.*)* | ______2____ | _____________ |
|  | **WHEN and HOW MUCH** |  |  |
| **8.** | Describe the number of times the intervention was delivered and over what period of time including the number of sessions, their schedule, and their duration, intensity or dose.  *In two visits 1st visit, 2nd visit (1month after)*  *The schedule, and duration, intensity or dose of intervention:* | _______3___ | _____________ |
|  | **TAILORING** |  |  |
| **9.** | If the intervention was planned to be personalised, titrated or adapted, then describe what, why, when, and how. | _______?_____ | _____________ |
|  | **MODIFICATIONS** |  |  |
| **10.^ǂ^** | If the intervention was modified during the course of the study, describe the changes (what, why, when, and how). | _____?____ | _____________ |
|  | **HOW WELL** |  |  |
| **11.** | Planned: If intervention adherence or fidelity was assessed, describe how and by whom, and if any strategies were used to maintain or improve fidelity, describe them. | ____?_____ | _____________ |
| **12.^ǂ^** | Actual: If intervention adherence or fidelity was assessed, describe the extent to which the intervention was delivered as planned: | _____?__ | _____________ |

** **Authors** - use N/A if an item is not applicable for the intervention being described. **Reviewers** – use ‘?’ if information about the element is not reported/not sufficiently reported.

† If the information is not provided in the primary paper, give details of where this information is available. This may include locations such as a published protocol or other published papers (provide citation details) or a website (provide the URL).

ǂ If completing the TIDieR checklist for a protocol, these items are not relevant to the protocol and cannot be described until the study is complete.

* We strongly recommend using this checklist in conjunction with the TIDieR guide (see *BMJ* 2014;348:g1687) which contains an explanation and elaboration for each item.

* The focus of TIDieR is on reporting details of the intervention elements (and where relevant, comparison elements) of a study. Other elements and methodological features of studies are covered by other reporting statements and checklists and have not been duplicated as part of the TIDieR checklist. When a **randomised trial** is being reported, the TIDieR checklist should be used in conjunction with the CONSORT statement (see [www.consort-statement.org](http://www.consort-statement.org)) as an extension of **Item 5 of the CONSORT 2010 Statement.** When a **clinical trial** **protocol** is being reported, the TIDieR checklist should be used in conjunction with the SPIRIT statement as an extension of **Item 11 of the SPIRIT 2013 Statement** (see [www.spirit-statement.org](http://www.spirit-statement.org)). For alternate study designs, TIDieR can be used in conjunction with the appropriate checklist for that study design (see [www.equator-network.org](http://www.equator-network.org)).
