# Supplementary material for: Exploring the contextual factors, behaviour change techniques, barriers and facilitators of interventions to improve oral health in people with severe mental illness: A qualitative study
Source: Front Psychiatry. 2022 Oct 11;13:971328. doi: 10.3389/fpsyt.2022.971328 (PMC9592713; doi:10.3389/fpsyt.2022.971328)
Supplement: Supplementary file 3 [file Table_3.DOCX]

**
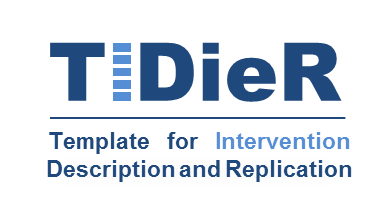
The TIDieR (Template for Intervention Description and Replication) Checklist*:**

Information to include when describing an intervention and the location of the information

| **Item number** | **Item: Almomani 2006** | **Where located **** | |
| --- | --- | --- | --- |
|  |  | Primary paper  (page or appendix  number) | Other ^†^ (details) |
|  | **BRIEF NAME** | 276 |  |
| **1.** | Provide the name or a phrase that describes the intervention.  *“dental education, oral hygiene instructions, and a tooth brushing reminding system (reminder post cards)”* | ________ | ______________ |
|  | **WHY** | 276 |  |
| **2.** | Describe any rationale, theory, or goal of the elements essential to the intervention.  *“Accordingly, the rationale of this study is based on previous studies which have demonstrated the effect of skill training programs for people with serious mental illness and the effect of oral health promotion programs in other populations.”* | ____________ | _____________ |
|  | **WHAT** | ? |  |
| **3.** | Materials: Describe any physical or informational materials used in the intervention, including those provided to participants or used in intervention delivery or in training of intervention providers. Provide information on where the materials can be accessed (e.g. online appendix, URL).  *“In the education room, a senior dental hygiene student educated each participant individually about the effects of chronic mental illness on oral health, advantages of good oral hygiene, and disadvantages of bad oral hygiene. The verbal presentation was supported by visuals from an educational flip chart and a handout. Also, the oral hygienist gave each participant two pamphlets to take home. The first pamphlet explained the impact of psychiatric disabilities, particularly medications, on oral health and the second pamphlet described the correct way of tooth brushing using a mechanical tooth- brush. After 4 weeks, all the participants received a handout with information about free dental services in the area.”*  Materials described as per copied text, however materials (e.g. presentation, pamphlets etc not available) | ___________    277-8 | _____________ |
| *4.* | Procedures: Describe each of the procedures, activities, and/or processes used in the intervention, including any enabling or support activities. As above, plus:  “*After the education, the dental hygienist gave every participant in Group A a mechanical toothbrush and instructed the participants to brush with the mechanical toothbrush twice a day for 2 minutes using the manufacturers’ instructions. Specifically, these instructions are “to brush with the powered toothbrush as you would normally brush with a manual toothbrush” and “be sure to brush all the surfaces.* *After the instruction, the dental hygienist gave the participants of group A a small plastic box and especially de- signed reminder post it notes with 60 pages. Participants were instructed to pull a paper each time they brushed their teeth and put the paper in the small plastic box. After one month (at post testing) the participant was asked to bring the small box to the examiner. Also, the dental hygienist contacted the participants in group A by tele- phone once a week to provide positive feedback and to reinforce the study instructions over the four-week study*.” | ___________ | _____________ |
|  | **WHO PROVIDED** |  |  |
| **5.** | For each category of intervention provider (e.g. psychologist, nursing assistant), describe their expertise, background and any specific training given.  *“senior dental hygiene student*”  Some expertise implied in role, however no mention of training/years of experience etc | ?  ___________ | _____________ |
|  | **HOW** | 277-8 |  |
| **6.** | Describe the modes of delivery (e.g. face-to-face or by some other mechanism, such as internet or telephone) of the intervention and whether it was provided individually or in a group.  *"In the education room, a senior dental hygiene student educated each participant individually"*  Also follow up reinforcement over telephone. | ___________ | _____________ |
|  | **WHERE** |  |  |
| **7.** | Describe the type(s) of location(s) where the intervention occurred, including any necessary infrastructure or relevant features.  *“All of the intervention strategies took place in Wyndott Center for Community and Behavioral Health, Kansas City, Kansas and all the partici- pants were consumers in the same center.”* Education room used for dental education | 277  ___________ | _____________ |
|  | **WHEN and HOW MUCH** |  |  |
| **8.** | Describe the number of times the intervention was delivered and over what period of time including the number of sessions, their schedule, and their duration, intensity or dose.  “*Each education session lasted for fifteen minutes... The instruction session lasted for ten minutes…. Also, the dental hygienist contacted the participants in group A by tele- phone once a week to provide positive feedback and to reinforce the study instructions over the four-week study.”* | 277-8  ___________ | _____________ |
|  | **TAILORING** |  |  |
| **9.** | If the intervention was planned to be personalised, titrated or adapted, then describe what, why, when, and how. | N/A  ___________ | _____________ |
|  | **MODIFICATIONS** |  |  |
| **10.^ǂ^** | If the intervention was modified during the course of the study, describe the changes (what, why, when, and how). | N/A  ___________ | _____________ |
|  | **HOW WELL** |  |  |
| **11.** | Planned: If intervention adherence or fidelity was assessed, describe how and by whom, and if any strategies were used to maintain or improve fidelity, describe them.  Not reported | ?  _________ | _____________ |
| **12.^ǂ^** | Actual: If intervention adherence or fidelity was assessed, describe the extent to which the intervention was delivered as planned.  *“Eight participants dropped out of the study and were explained through phone calls that they did not complete the study because of personal reasons (i.e. moving, hospitalization). No one dropped out of the study because he/she did not like the intervention.”* | 278_____ | _____________ |

** **Authors** - use N/A if an item is not applicable for the intervention being described. **Reviewers** – use ‘?’ if information about the element is not reported/not sufficiently reported.

† If the information is not provided in the primary paper, give details of where this information is available. This may include locations such as a published protocol or other published papers (provide citation details) or a website (provide the URL).

ǂ If completing the TIDieR checklist for a protocol, these items are not relevant to the protocol and cannot be described until the study is complete.

* We strongly recommend using this checklist in conjunction with the TIDieR guide (see *BMJ* 2014;348:g1687) which contains an explanation and elaboration for each item.

* The focus of TIDieR is on reporting details of the intervention elements (and where relevant, comparison elements) of a study. Other elements and methodological features of studies are covered by other reporting statements and checklists and have not been duplicated as part of the TIDieR checklist. When a **randomised trial** is being reported, the TIDieR checklist should be used in conjunction with the CONSORT statement (see [www.consort-statement.org](http://www.consort-statement.org)) as an extension of **Item 5 of the CONSORT 2010 Statement.** When a **clinical trial** **protocol** is being reported, the TIDieR checklist should be used in conjunction with the SPIRIT statement as an extension of **Item 11 of the SPIRIT 2013 Statement** (see [www.spirit-statement.org](http://www.spirit-statement.org)). For alternate study designs, TIDieR can be used in conjunction with the appropriate checklist for that study design (see [www.equator-network.org](http://www.equator-network.org)).
