# Supplementary material for: Exploring the contextual factors, behaviour change techniques, barriers and facilitators of interventions to improve oral health in people with severe mental illness: A qualitative study
Source: Front Psychiatry. 2022 Oct 11;13:971328. doi: 10.3389/fpsyt.2022.971328 (PMC9592713; doi:10.3389/fpsyt.2022.971328)
Supplement: Supplementary file 4 [file Table_4.DOCX]

**
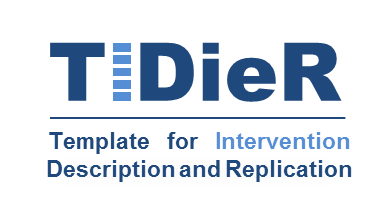
The TIDieR (Template for Intervention Description and Replication) Checklist*:**

Information to include when describing an intervention and the location of the information

| **Item number** | **Item: Almomani 2009** | **Where located **** | |
| --- | --- | --- | --- |
|  |  | Primary paper  (page or appendix  number) | Other ^†^ (details) |
|  | **BRIEF NAME** | 648 |  |
| **1.** | Provide the name or a phrase that describes the intervention.  “…*brief MI session immediately before an oral health education* session” | ________ | ______________ |
|  | **WHY** | 648 |  |
| **2.** | Describe any rationale, theory, or goal of the elements essential to the intervention.  *“Although dental health education has positive effects on plaque accumulation and knowledge level, effects are relatively weak and may be short-lived. The effects of educational interventions may be improved by enhancing individuals’ motivation to implement and maintain behavioral changes. Motivational interviewing (MI) is effective in enhancing motivation for changing a wide range of health behaviors, including oral hygiene. MI uses specific methods to elicit the individual’s “internal” motivation to*  *foster autonomous self-regulation, rather than the practitioner providing “external” reasons to persuade the individual to change. The purpose of this study was to investigate whether providing a brief MI session immediately before an oral health education session would enhance the education effect. It was hypothesized that MI plus oral health education would reduce plaque scores and improve knowledge and autonomous self-regulation more than would oral health education alone”* | ___________ | _____________ |
|  | **WHAT** | ? |  |
| **3.** | Materials: Describe any physical or informational materials used in the intervention, including those provided to participants or used in intervention delivery or in training of intervention providers. Provide information on where the materials can be accessed (e.g. online appendix, URL).  Some aspects of intervention described, e.g. education component involved pamphlets and reminder system. However specific materials not available, and elements of MI not described (e.g. questions used). | ___________    649 | _____________ |
| *4.* | Procedures: Describe each of the procedures, activities, and/or processes used in the intervention, including any enabling or support activities.  *“The brief MI sessions (15-20 min) were conducted by a doctoral psychology student trained in MI and supervised by an expert in MI (co-author DC) ….. For those in the MI arm, the intervention was conducted prior to the education session and focused on exploring advantages and disadvantages, motivation and confidence, and personal values related to daily toothbrushing and oral health. The principal researcher administered the education sessions, providing information about the effects of severe mental illness on oral health, the advantages of good oral hygiene, and the disadvantages of bad oral hygiene. All participants received two pamphlets summarizing the information from the education session, instruction in using a mechanical toothbrush (Crest Spin Brush Pro), a reminder system, and weekly telephone calls (for 4 wks).”* | ___________ | _____________ |
|  | **WHO PROVIDED** |  |  |
| **5.** | For each category of intervention provider (e.g. psychologist, nursing assistant), describe their expertise, background and any specific training given.  MI: *“conducted by a doctoral psychology student trained in MI and supervised by an expert in MI (co-author DC)”* and education “*principal researcher administered the education sessions*”  Some expertise implied in role, however no mention of training/years of experience etc | ?  ___________ | _____________ |
|  | **HOW** | ? |  |
| **6.** | Describe the modes of delivery (e.g. face-to-face or by some other mechanism, such as internet or telephone) of the intervention and whether it was provided individually or in a group.  Assumption that MI is individual but this is not explicitly stated. Education it is not clear whether it is individual or group. | ___________ | _____________ |
|  | **WHERE** |  |  |
| **7.** | Describe the type(s) of location(s) where the intervention occurred, including any necessary infrastructure or relevant features.  *“recruited from a community program”* but details of general setting or where the intervention took place. | ?  ___________ | _____________ |
|  | **WHEN and HOW MUCH** |  |  |
| **8.** | Describe the number of times the intervention was delivered and over what period of time including the number of sessions, their schedule, and their duration, intensity or dose.  “*MI sessions (15-20 min)”* however, no information on length of education intervention or how long afterwards it took place. | ?  ___________ | _____________ |
|  | **TAILORING** |  |  |
| **9.** | If the intervention was planned to be personalised, titrated or adapted, then describe what, why, when, and how. | N/A  ___________ | _____________ |
|  | **MODIFICATIONS** |  |  |
| **10.^ǂ^** | If the intervention was modified during the course of the study, describe the changes (what, why, when, and how). | N/A  ___________ | _____________ |
|  | **HOW WELL** |  |  |
| **11.** | Planned: If intervention adherence or fidelity was assessed, describe how and by whom, and if any strategies were used to maintain or improve fidelity, describe them.  *“The brief MI sessions (15-20 min) were conducted by a doctoral psychology student trained in MI and supervised by an expert in MI (co-author DC), who ensured fidelity by listening to audio- tapes of the sessions, rating performance, and providing feedback”* | 649  _________ | _____________ |
| **12.^ǂ^** | Actual: If intervention adherence or fidelity was assessed, describe the extent to which the intervention was delivered as planned. | ?  _________ | _____________ |

** **Authors** - use N/A if an item is not applicable for the intervention being described. **Reviewers** – use ‘?’ if information about the element is not reported/not sufficiently reported.

† If the information is not provided in the primary paper, give details of where this information is available. This may include locations such as a published protocol or other published papers (provide citation details) or a website (provide the URL).

ǂ If completing the TIDieR checklist for a protocol, these items are not relevant to the protocol and cannot be described until the study is complete.

* We strongly recommend using this checklist in conjunction with the TIDieR guide (see *BMJ* 2014;348:g1687) which contains an explanation and elaboration for each item.

* The focus of TIDieR is on reporting details of the intervention elements (and where relevant, comparison elements) of a study. Other elements and methodological features of studies are covered by other reporting statements and checklists and have not been duplicated as part of the TIDieR checklist. When a **randomised trial** is being reported, the TIDieR checklist should be used in conjunction with the CONSORT statement (see [www.consort-statement.org](http://www.consort-statement.org)) as an extension of **Item 5 of the CONSORT 2010 Statement.** When a **clinical trial** **protocol** is being reported, the TIDieR checklist should be used in conjunction with the SPIRIT statement as an extension of **Item 11 of the SPIRIT 2013 Statement** (see [www.spirit-statement.org](http://www.spirit-statement.org)). For alternate study designs, TIDieR can be used in conjunction with the appropriate checklist for that study design (see [www.equator-network.org](http://www.equator-network.org)).
