# Supplementary material for: Exploring the contextual factors, behaviour change techniques, barriers and facilitators of interventions to improve oral health in people with severe mental illness: A qualitative study
Source: Front Psychiatry. 2022 Oct 11;13:971328. doi: 10.3389/fpsyt.2022.971328 (PMC9592713; doi:10.3389/fpsyt.2022.971328)
Supplement: Supplementary file 5 [file Table_5.DOCX]

**
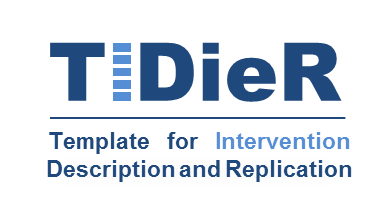
The TIDieR (Template for Intervention Description and Replication) Checklist*:**

Information to include when describing an intervention and the location of the information

| **Item number** | **Item: de Mey 2016** | **Where located **** | |
| --- | --- | --- | --- |
|  |  | Primary paper  (page or appendix  number) | Other ^†^ (details) |
|  | **BRIEF NAME** | 194 (abstract) |  |
| **1.** | Provide the name or a phrase that describes the intervention.  *“an educational intervention about oral hygiene on the knowledge of mental health nurses, and …. the effects of an oral care intervention on oral health in patients with severe mental illness*” | ________ | ______________ |
|  | **WHY** | ?/195 |  |
| **2.** | Describe any rationale, theory, or goal of the elements essential to the intervention.  “*Since mental health staff consisting of nurses, student nurses, and social workers often have relatively frequent and intensive contact with SMI patients, they could play a major role in the oral health care of these patients… In cooperation with dentists and dental hygienists, nurses have the opportunity to integrate oral health care into everyday psychiatric treatment and care in order to help their patients maintain oral health”* | ___________ | _____________ |
|  | **WHAT** |  |  |
| **3.** | Materials: Describe any physical or informational materials used in the intervention, including those provided to participants or used in intervention delivery or in training of intervention providers. Provide information on where the materials can be accessed (e.g. online appendix, URL).  *20 minute PowerPoint presentation, cleaning tools (mouthwash, tongue cleaner, interdental cleaning aids) for demonstration*  *Soft toothbrush, fluoridated toothpaste, brushing instruction card, images of toothbrush in the mouth. Also reference to patient and nurse keeping a log, but no reference to if a “logbook” was provided* | ?/196  __________  196 | _____________ |
| *4.* | Procedures: Describe each of the procedures, activities, and/or processes used in the intervention, including any enabling or support activities.  *PowerPoint presentation for nurses, with explanations based on images and text. Demonstration to nurses using cleaning tools.*  *Patient provided with brush and toothpaste, given instructions (examples of method used, specific instructions and the technique the teaching was based on – Bass method are all described). Patients and nurses asked to keep log book and nurses asked to give instruction.* | ___________ | _____________ |
|  | **WHO PROVIDED** |  |  |
| **5.** | For each category of intervention provider (e.g. psychologist, nursing assistant), describe their expertise, background and any specific training given.  Presentation done by two oral hygienist/researcher. Oral care plan done by oral hygienist  Some expertise implied in role, however no mention of training/years of experience etc | ?/196  ___________ | _____________ |
|  | **HOW** | ?/196 |  |
| **6.** | Describe the modes of delivery (e.g. face-to-face or by some other mechanism, such as internet or telephone) of the intervention and whether it was provided individually or in a group.  *Implied group intervention and face to face for PowerPoint but not explicitly stated, e.g. The short duration of the presentation meant it was possible to incorporate it into the daily routines of the participating treatment settings*  *Patient intervention provided after oral examination, so implied face to face and individual, but again not explicitly stated* | ___________ | _____________ |
|  | **WHERE** |  |  |
| **7.** | Describe the type(s) of location(s) where the intervention occurred, including any necessary infrastructure or relevant features.  *The study was carried out in a large mental health organization in the western part of the Netherlands.*  *Three departments for long-term care for adults and elderly patients participated in this study, both inpatients and outpatients.*  *For practical reasons, the pretest and posttest measurements were performed in the treatment locations of the patients using a regular chair (no dental chair)* | 195-196  ___________ | _____________ |
|  | **WHEN and HOW MUCH** |  |  |
| **8.** | *Describe the number of times the intervention was delivered and over what period of time including the number of sessions, their schedule, and their duration, intensity or dose.*  *One PowerPoint presentation for 20 minutes. No reporting of time patients were instructed on brushing technique. Patients and nurses asked to keep log of daily mouthcare activities for 4 weeks.* | ?/196  ___________ | _____________ |
|  | **TAILORING** |  |  |
| **9.** | If the intervention was planned to be personalised, titrated or adapted, then describe what, why, when, and how. | N/A  ___________ | _____________ |
|  | **MODIFICATIONS** |  |  |
| **10.^ǂ^** | If the intervention was modified during the course of the study, describe the changes (what, why, when, and how). | N/A  ___________ | _____________ |
|  | **HOW WELL** |  |  |
| **11.** | Planned: If intervention adherence or fidelity was assessed, describe how and by whom, and if any strategies were used to maintain or improve fidelity, describe them.  *“Commitment and compliance with the oral treatment plan were encouraged by asking the patient and the accompanying nurse to keep a log so that daily mouth care activities could be monitored”* | ?/196  _________ | _____________ |
| **12.^ǂ^** | Actual: If intervention adherence or fidelity was assessed, describe the extent to which the intervention was delivered as planned.  *“Three patients were lost to follow-up because they moved to another institution”*  *“A limitation of this pilot study is that it did not systematically monitor and register the nurses’ activities with regard to their patients’ oral health care, which limited the possibility of tailored supervision and support for nurses in order to improve intervention fidelity”* | ?/196+198 | _____________ |

** **Authors** - use N/A if an item is not applicable for the intervention being described. **Reviewers** – use ‘?’ if information about the element is not reported/not sufficiently reported.

† If the information is not provided in the primary paper, give details of where this information is available. This may include locations such as a published protocol or other published papers (provide citation details) or a website (provide the URL).

ǂ If completing the TIDieR checklist for a protocol, these items are not relevant to the protocol and cannot be described until the study is complete.

* We strongly recommend using this checklist in conjunction with the TIDieR guide (see *BMJ* 2014;348:g1687) which contains an explanation and elaboration for each item.

* The focus of TIDieR is on reporting details of the intervention elements (and where relevant, comparison elements) of a study. Other elements and methodological features of studies are covered by other reporting statements and checklists and have not been duplicated as part of the TIDieR checklist. When a **randomised trial** is being reported, the TIDieR checklist should be used in conjunction with the CONSORT statement (see [www.consort-statement.org](http://www.consort-statement.org)) as an extension of **Item 5 of the CONSORT 2010 Statement.** When a **clinical trial** **protocol** is being reported, the TIDieR checklist should be used in conjunction with the SPIRIT statement as an extension of **Item 11 of the SPIRIT 2013 Statement** (see [www.spirit-statement.org](http://www.spirit-statement.org)). For alternate study designs, TIDieR can be used in conjunction with the appropriate checklist for that study design (see [www.equator-network.org](http://www.equator-network.org)).
