# Supplementary material for: Exploring the contextual factors, behaviour change techniques, barriers and facilitators of interventions to improve oral health in people with severe mental illness: A qualitative study
Source: Front Psychiatry. 2022 Oct 11;13:971328. doi: 10.3389/fpsyt.2022.971328 (PMC9592713; doi:10.3389/fpsyt.2022.971328)
Supplement: Supplementary file 7 [file Table_7.DOCX]

**
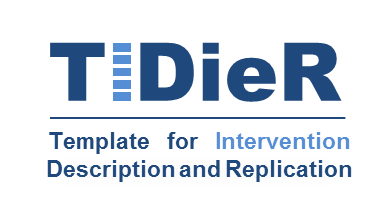
The TIDieR (Template for Intervention Description and Replication) Checklist*:**

Information to include when describing an intervention and the location of the information

| **Item number** | **Item: Jean** | **Where located **** | |
| --- | --- | --- | --- |
|  |  | Primary paper  (page or appendix  number) | Other ^†^ (details) |
|  | **BRIEF NAME** | 865 |  |
| **1.** | Provide the name or a phrase that describes the intervention.  *Mangosteen fruit an adjunctive to scaling and root planning* | ________ | ______________ |
|  | **WHY** | 864-865 |  |
| **2.** | Describe any rationale, theory, or goal of the elements essential to the intervention.  *Several studies show that Mangosteen is rich in xanthones*  *that has antioxidant, anti‑inflammatory, anti-allergy,*  *antibacterial, anticancer, and antifungal effects [12].*  *Previous cross-sectional as well as longitudinal research*  *suggests that diet may play a role in preventing*  *periodontal disease [13] . In addition, high-fibre foods,*  *specifically fruit and grains, reduce periodontal disease*  *progression, especially among older adults [14]. A pilot*  *intervention study by Kondo, K. et al among 21*  *participants shows that high-fibre diets improve*  *periodontal disease in high-risk subjects [15].* | ___________ | _____________ |
|  | **WHAT** | 865 |  |
| **3.** | Materials: Describe any physical or informational materials used in the intervention, including those provided to participants or used in intervention delivery or in training of intervention providers. Provide information on where the materials can be accessed (e.g. online appendix, URL).  *Sterile Gracey curettes (for scaling and root planning) & 200mg Mangosteen fruit per day. Diary for monitoring intake of mangosteen fruit* | ___________  865/? | _____________ |
| *4.* | Procedures: Describe each of the procedures, activities, and/or processes used in the intervention, including any enabling or support activities. Basic description as below (not detailed in relation to scaling does describe process)  *Complete supragingival Ultra Sonic Scaling followed by thorough subgingival Scaling and Root Planning was done using sterile Gracey curettes for all the patients. Group II*  *(test group) patients were provided with mangosteen fruit (approximately 200mg per day) and were instructed to consume it twice daily as a whole fruit without the addition of any other ingredients until the study period and each patient was monitored. Dietary habits were not changed in both groups. A diary was maintained to note the daily intake of mangosteen fruit during the entire study*  *period.* | ___________ | _____________ |
|  | **WHO PROVIDED** |  |  |
| **5.** | For each category of intervention provider (e.g. psychologist, nursing assistant), describe their expertise, background and any specific training given.  Not reported | ?  ___________ | _____________ |
|  | **HOW** | ?/865 |  |
| **6.** | Describe the modes of delivery (e.g. face-to-face or by some other mechanism, such as internet or telephone) of the intervention and whether it was provided individually or in a group.  Not reported but given nature of intervention, would imply it has to be done individually and face to face (due to scaling part of intervention), although ensuring consumption of mangosteen fruit could be done through another mechanism | ___________ | _____________ |
|  | **WHERE** |  |  |
| **7.** | Describe the type(s) of location(s) where the intervention occurred, including any necessary infrastructure or relevant features.  Patients appear to be recruited from Dr. Fernandez home for schizophrenia but no details on where intervention occurred | ?  ___________ | _____________ |
|  | **WHEN and HOW MUCH** |  |  |
| **8.** | Describe the number of times the intervention was delivered and over what period of time including the number of sessions, their schedule, and their duration, intensity or dose. Mangosteen fruit consumed twice daily (200mg in total per day) throughout 3 month study period. SRP done prior to fruit, not explicitly stated but implied this is only done once | 865/?  ___________ | _____________ |
|  | **TAILORING** |  |  |
| **9.** | If the intervention was planned to be personalised, titrated or adapted, then describe what, why, when, and how. | N/A  ___________ | _____________ |
|  | **MODIFICATIONS** |  |  |
| **10.^ǂ^** | If the intervention was modified during the course of the study, describe the changes (what, why, when, and how). | N/A  ___________ | _____________ |
|  | **HOW WELL** |  |  |
| **11.** | Planned: If intervention adherence or fidelity was assessed, describe how and by whom, and if any strategies were used to maintain or improve fidelity, describe them.  A diary was maintained to note the daily intake of mangosteen fruit during the entire study period | ?/865  _________ | _____________ |
| **12.^ǂ^** | Actual: If intervention adherence or fidelity was assessed, describe the extent to which the intervention was delivered as planned. | ?  _________ | _____________ |

** **Authors** - use N/A if an item is not applicable for the intervention being described. **Reviewers** – use ‘?’ if information about the element is not reported/not sufficiently reported.

† If the information is not provided in the primary paper, give details of where this information is available. This may include locations such as a published protocol or other published papers (provide citation details) or a website (provide the URL).

ǂ If completing the TIDieR checklist for a protocol, these items are not relevant to the protocol and cannot be described until the study is complete.

* We strongly recommend using this checklist in conjunction with the TIDieR guide (see *BMJ* 2014;348:g1687) which contains an explanation and elaboration for each item.

* The focus of TIDieR is on reporting details of the intervention elements (and where relevant, comparison elements) of a study. Other elements and methodological features of studies are covered by other reporting statements and checklists and have not been duplicated as part of the TIDieR checklist. When a **randomised trial** is being reported, the TIDieR checklist should be used in conjunction with the CONSORT statement (see [www.consort-statement.org](http://www.consort-statement.org)) as an extension of **Item 5 of the CONSORT 2010 Statement.** When a **clinical trial** **protocol** is being reported, the TIDieR checklist should be used in conjunction with the SPIRIT statement as an extension of **Item 11 of the SPIRIT 2013 Statement** (see [www.spirit-statement.org](http://www.spirit-statement.org)). For alternate study designs, TIDieR can be used in conjunction with the appropriate checklist for that study design (see [www.equator-network.org](http://www.equator-network.org)).
