# Supplementary material for: Exploring the contextual factors, behaviour change techniques, barriers and facilitators of interventions to improve oral health in people with severe mental illness: A qualitative study
Source: Front Psychiatry. 2022 Oct 11;13:971328. doi: 10.3389/fpsyt.2022.971328 (PMC9592713; doi:10.3389/fpsyt.2022.971328)
Supplement: Supplementary file 9 [file Table_9.DOCX]

**
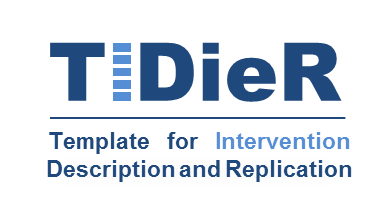
The TIDieR (Template for Intervention Description and Replication) Checklist*:**

Information to include when describing an intervention and the location of the information

| **Item number** | **Item: Kuo 2020** | **Where located **** | |
| --- | --- | --- | --- |
|  |  | Primary paper  (page or appendix  number) | Other ^†^ (details) |
|  | **BRIEF NAME** | 3 |  |
| **1.** | Provide the name or a phrase that describes the intervention.  “The oral health promotion program was a composite intervention with both group and individual components” | ________ | ______________ |
|  | **WHY** | 2 |  |
| **2.** | Describe any rationale, theory, or goal of the elements essential to the intervention.  *“*Khokhar et al. conducted a systematic review to explore the effects of oral health education on people with serious mental illness. They concluded that there was no evidence that oral health advice helped those with SMI in terms of clinically meaningful outcomes [26]. Furthermore, they suggested that more good-quality studies are needed to obtain concrete evidence to aid in decision-making about the effectiveness of oral health interventions for those with SMI. Another study recommended that there be further research on the efects of more comprehensive oral health promotion programs with a non-intervention group [23]. To address this issue, we therefore designed a cluster randomized controlled trial to determine the effects of a composite oral health promotion program, including individual and group components, on patients with SMI” | ___________ | _____________ |
|  | **WHAT** | ?/3 |  |
| **3.** | Materials: Describe any physical or informational materials used in the intervention, including those provided to participants or used in intervention delivery or in training of intervention providers. Provide information on where the materials can be accessed (e.g. online appendix, URL).  *Educational materials (content described, but format not reported and materials not provided), pictures of Bass technique, songs around brushing (no further information given), reward tokens* | ___________  3 | _____________ |
| *4.* | Procedures: Describe each of the procedures, activities, and/or processes used in the intervention, including any enabling or support activities.  Group education over several sessions, each followed by individual brushing technique sessions, displaying images in bathrooms, reminder songs played, “behavioural modification” using tokens | ___________ | _____________ |
|  | **WHO PROVIDED** |  |  |
| **5.** | For each category of intervention provider (e.g. psychologist, nursing assistant), describe their expertise, background and any specific training given.  “*Before the study, nurses in the ward selected as the intervention group received training in an accurate Bass tooth-brushing technique from the dentist, and in basic behavioural modification methods from a clinical psychologist”.  “The group education session was conducted by trained nurses”*  *“…training in the Bass toothbrushing technique by trained nurses”* | 3  ___________ | _____________ |
|  | **HOW** | 3 |  |
| **6.** | Describe the modes of delivery (e.g. face-to-face or by some other mechanism, such as internet or telephone) of the intervention and whether it was provided individually or in a group.  *Group education provided by nurses (implied face to face), followed by individual brushing technique instructions.* | ___________ | _____________ |
|  | **WHERE** |  |  |
| **7.** | Describe the type(s) of location(s) where the intervention occurred, including any necessary infrastructure or relevant features.  *“This study was conducted in the psychiatric wards of a general hospital in central Taiwan. The hospital provides 4 psychiatric wards to serve 300 chronic psychiatric patients (75 patients in each ward).”* | 2  ___________ | _____________ |
|  | **WHEN and HOW MUCH** |  |  |
| **8.** | Describe the number of times the intervention was delivered and over what period of time including the number of sessions, their schedule, and their duration, intensity or dose. Group sessions – five in total (60 minutes each).  Individual sessions – five in total (after each group session), no time provided  Songs – Five times each day  12 week experimental study | 3  ___________ | _____________ |
|  | **TAILORING** |  |  |
| **9.** | If the intervention was planned to be personalised, titrated or adapted, then describe what, why, when, and how. | N/A  ___________ | _____________ |
|  | **MODIFICATIONS** |  |  |
| **10.^ǂ^** | If the intervention was modified during the course of the study, describe the changes (what, why, when, and how). | N/A  ___________ | _____________ |
|  | **HOW WELL** |  |  |
| **11.** | Planned: If intervention adherence or fidelity was assessed, describe how and by whom, and if any strategies were used to maintain or improve fidelity, describe them. | ?  _________ | _____________ |
| **12.^ǂ^** | Actual: If intervention adherence or fidelity was assessed, describe the extent to which the intervention was delivered as planned. | ?  _________ | _____________ |

** **Authors** - use N/A if an item is not applicable for the intervention being described. **Reviewers** – use ‘?’ if information about the element is not reported/not sufficiently reported.

† If the information is not provided in the primary paper, give details of where this information is available. This may include locations such as a published protocol or other published papers (provide citation details) or a website (provide the URL).

ǂ If completing the TIDieR checklist for a protocol, these items are not relevant to the protocol and cannot be described until the study is complete.

* We strongly recommend using this checklist in conjunction with the TIDieR guide (see *BMJ* 2014;348:g1687) which contains an explanation and elaboration for each item.

* The focus of TIDieR is on reporting details of the intervention elements (and where relevant, comparison elements) of a study. Other elements and methodological features of studies are covered by other reporting statements and checklists and have not been duplicated as part of the TIDieR checklist. When a **randomised trial** is being reported, the TIDieR checklist should be used in conjunction with the CONSORT statement (see [www.consort-statement.org](http://www.consort-statement.org)) as an extension of **Item 5 of the CONSORT 2010 Statement.** When a **clinical trial** **protocol** is being reported, the TIDieR checklist should be used in conjunction with the SPIRIT statement as an extension of **Item 11 of the SPIRIT 2013 Statement** (see [www.spirit-statement.org](http://www.spirit-statement.org)). For alternate study designs, TIDieR can be used in conjunction with the appropriate checklist for that study design (see [www.equator-network.org](http://www.equator-network.org)).
