# Supplementary material for: Exploring the contextual factors, behaviour change techniques, barriers and facilitators of interventions to improve oral health in people with severe mental illness: A qualitative study
Source: Front Psychiatry. 2022 Oct 11;13:971328. doi: 10.3389/fpsyt.2022.971328 (PMC9592713; doi:10.3389/fpsyt.2022.971328)
Supplement: Supplementary file 11 [file Table_11.DOCX]

**
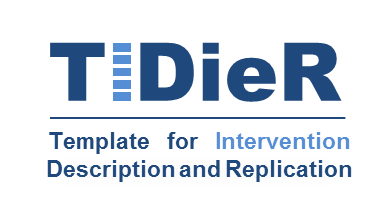
The TIDieR (Template for Intervention Description and Replication) Checklist*:**

Information to include when describing an intervention and the location of the information

| **Item number** | **Item: Nurbaya 2020** | **Where located **** | |
| --- | --- | --- | --- |
|  |  | Primary paper  (page or appendix  number) | Other ^†^ (details) |
|  | **BRIEF NAME** | 942 |  |
| **1.** | Provide the name or a phrase that describes the intervention.  “education and training teeth brushing” | ________ | ______________ |
|  | **WHY** | ?/942 |  |
| **2.** | Describe any rationale, theory, or goal of the elements essential to the intervention.  *Some justification but not very detailed* | ___________ | _____________ |
|  | **WHAT** | ? |  |
| **3.** | Materials: Describe any physical or informational materials used in the intervention, including those provided to participants or used in intervention delivery or in training of intervention providers. Provide information on where the materials can be accessed (e.g. online appendix, URL).  *Educational materials not described* | ___________  ?/942 | _____________ |
| *4.* | Procedures: Describe each of the procedures, activities, and/or processes used in the intervention, including any enabling or support activities.  Brief description “Education 2 times with a distance of 1 week, how to brush the tooth modified Bass in the dental model as well as training teeth brushing by the patient.” | ___________ | _____________ |
|  | **WHO PROVIDED** |  |  |
| **5.** | For each category of intervention provider (e.g. psychologist, nursing assistant), describe their expertise, background and any specific training given.  Not reported | ?  ___________ | _____________ |
|  | **HOW** | ? |  |
| **6.** | Describe the modes of delivery (e.g. face-to-face or by some other mechanism, such as internet or telephone) of the intervention and whether it was provided individually or in a group.  Not reported | ___________ | _____________ |
|  | **WHERE** |  |  |
| **7.** | Describe the type(s) of location(s) where the intervention occurred, including any necessary infrastructure or relevant features.  Patients appear to be recruited from Mental Hospital Soeprapto Propinsi Bengkulu (MHSPB) but no details on where intervention occurred | ?  ___________ | _____________ |
|  | **WHEN and HOW MUCH** |  |  |
| **8.** | Describe the number of times the intervention was delivered and over what period of time including the number of sessions, their schedule, and their duration, intensity or dose.  Appears education was delivered twice, one week apart, but no further detail on how long they lasted etc | ?/942  ___________ | _____________ |
|  | **TAILORING** |  |  |
| **9.** | If the intervention was planned to be personalised, titrated or adapted, then describe what, why, when, and how. | N/A  ___________ | _____________ |
|  | **MODIFICATIONS** |  |  |
| **10.^ǂ^** | If the intervention was modified during the course of the study, describe the changes (what, why, when, and how). | N/A  ___________ | _____________ |
|  | **HOW WELL** |  |  |
| **11.** | Planned: If intervention adherence or fidelity was assessed, describe how and by whom, and if any strategies were used to maintain or improve fidelity, describe them. | ?  _________ | _____________ |
| **12.^ǂ^** | Actual: If intervention adherence or fidelity was assessed, describe the extent to which the intervention was delivered as planned. | ?  _________ | _____________ |

** **Authors** - use N/A if an item is not applicable for the intervention being described. **Reviewers** – use ‘?’ if information about the element is not reported/not sufficiently reported.

† If the information is not provided in the primary paper, give details of where this information is available. This may include locations such as a published protocol or other published papers (provide citation details) or a website (provide the URL).

ǂ If completing the TIDieR checklist for a protocol, these items are not relevant to the protocol and cannot be described until the study is complete.

* We strongly recommend using this checklist in conjunction with the TIDieR guide (see *BMJ* 2014;348:g1687) which contains an explanation and elaboration for each item.

* The focus of TIDieR is on reporting details of the intervention elements (and where relevant, comparison elements) of a study. Other elements and methodological features of studies are covered by other reporting statements and checklists and have not been duplicated as part of the TIDieR checklist. When a **randomised trial** is being reported, the TIDieR checklist should be used in conjunction with the CONSORT statement (see [www.consort-statement.org](http://www.consort-statement.org)) as an extension of **Item 5 of the CONSORT 2010 Statement.** When a **clinical trial** **protocol** is being reported, the TIDieR checklist should be used in conjunction with the SPIRIT statement as an extension of **Item 11 of the SPIRIT 2013 Statement** (see [www.spirit-statement.org](http://www.spirit-statement.org)). For alternate study designs, TIDieR can be used in conjunction with the appropriate checklist for that study design (see [www.equator-network.org](http://www.equator-network.org)).
