# Supplementary material for: Exploring the contextual factors, behaviour change techniques, barriers and facilitators of interventions to improve oral health in people with severe mental illness: A qualitative study
Source: Front Psychiatry. 2022 Oct 11;13:971328. doi: 10.3389/fpsyt.2022.971328 (PMC9592713; doi:10.3389/fpsyt.2022.971328)
Supplement: Supplementary file 12 [file Table_12.DOCX]

**
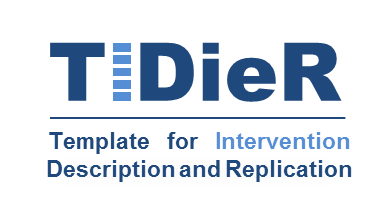
The TIDieR (Template for Intervention Description and Replication) Checklist*:**

Information to include when describing an intervention and the location of the information

| **Item number** | **Item: Singahl 2021** | **Where located **** | |
| --- | --- | --- | --- |
|  |  | Primary paper  (page or appendix  number) | Other ^†^ (details) |
|  | **BRIEF NAME** | 357 |  |
| **1.** | Provide the name or a phrase that describes the intervention*.*  *“….a combination of the use of a battery-operated toothbrush and a related educational intervention”* | ________ | ______________ |
|  | **WHY** | 358 |  |
| **2.** | Describe any rationale, theory, or goal of the elements essential to the intervention | ___________ | _____________ |
|  | **WHAT** | ?/3 |  |
| **3.** | Materials: Describe any physical or informational materials used in the intervention, including those provided to participants or used in intervention delivery or in training of intervention providers. Provide information on where the materials can be accessed (e.g. online appendix, URL).  *Calendar with stickers, “Crest cavity protection toothpaste”, a battery-operated Arm and Hammer Truly Radiant Spin Brush, Sun Star Gum ultrasoft manual toothbrush, video demonstrations on the modifed Bass toothbrushing technique (specific to toothbrush) – videos not provided* | ___________ | _____________ |
| *4.* | Procedures: Describe each of the procedures, activities, and/or processes used in the intervention, including any enabling or support activities.  Provision of toothbrushes and toothpaste, instructions and video demonstrations (for two groups), evaluation and feedback on oral hygiene and brushing technique, participants asked to complete calendar with stickers | ?/360  _________ | _____________ |
|  | **WHO PROVIDED** |  |  |
| **5** | For each category of intervention provider (e.g. psychologist, nursing assistant), describe their expertise, background and any specific training given.  *Dental hygiene students*  “*All students provided the participants with ten (10) min of evaluation and instruction per visit.*  *Prior to the start of the study, the students received three (3) hours of training regarding sensitivity towards people with a diagnosis of mental illness and dental fears of people with mental illness by professionals in the Department of Psychiatric Rehabilitation and Counseling Professions at*  *the Rutgers School of Health Professions”* | 360  ___________ | _____________ |
|  | **HOW** | ?/360 |  |
| **6.** | Describe the modes of delivery (e.g. face-to-face or by some other mechanism, such as internet or telephone) of the intervention and whether it was provided individually or in a group.  *Implied individual feedback, but little information provided* | ___________ | _____________ |
|  | **WHERE** |  |  |
| **7.** | Describe the type(s) of location(s) where the intervention occurred, including any necessary infrastructure or relevant features.  *“Participants were recruited from rural and urban outpatient mental wellness centers in central and northwestern New Jersey” – No details on where intervention was delivered* | ?  ___________ | _____________ |
|  | **WHEN and HOW MUCH** |  |  |
| **8.** | Describe the number of times the intervention was delivered and over what period of time including the number of sessions, their schedule, and their duration, intensity or dose. Instruction and feedback on brushing – 10 minutes, unsure if this was only done once or repeated.  No information on length of video demonstration. Length of study 4 weeks. | ?  ___________ | _____________ |
|  | **TAILORING** |  |  |
| **9.** | If the intervention was planned to be personalised, titrated or adapted, then describe what, why, when, and how. | N/A  ___________ | _____________ |
|  | **MODIFICATIONS** |  |  |
| **10.^ǂ^** | If the intervention was modified during the course of the study, describe the changes (what, why, when, and how). | N/A  ___________ | _____________ |
|  | **HOW WELL** |  |  |
| **11.** | Planned: If intervention adherence or fidelity was assessed, describe how and by whom, and if any strategies were used to maintain or improve fidelity, describe them. | ?  _________ | _____________ |
| **12.^ǂ^** | Actual: If intervention adherence or fidelity was assessed, describe the extent to which the intervention was delivered as planned.  “*Sixty-one participants (70.11%) returned the calendar”* | ?  _________ | _____________ |

** **Authors** - use N/A if an item is not applicable for the intervention being described. **Reviewers** – use ‘?’ if information about the element is not reported/not sufficiently reported.

† If the information is not provided in the primary paper, give details of where this information is available. This may include locations such as a published protocol or other published papers (provide citation details) or a website (provide the URL).

ǂ If completing the TIDieR checklist for a protocol, these items are not relevant to the protocol and cannot be described until the study is complete.

* We strongly recommend using this checklist in conjunction with the TIDieR guide (see *BMJ* 2014;348:g1687) which contains an explanation and elaboration for each item.

* The focus of TIDieR is on reporting details of the intervention elements (and where relevant, comparison elements) of a study. Other elements and methodological features of studies are covered by other reporting statements and checklists and have not been duplicated as part of the TIDieR checklist. When a **randomised trial** is being reported, the TIDieR checklist should be used in conjunction with the CONSORT statement (see [www.consort-statement.org](http://www.consort-statement.org)) as an extension of **Item 5 of the CONSORT 2010 Statement.** When a **clinical trial** **protocol** is being reported, the TIDieR checklist should be used in conjunction with the SPIRIT statement as an extension of **Item 11 of the SPIRIT 2013 Statement** (see [www.spirit-statement.org](http://www.spirit-statement.org)). For alternate study designs, TIDieR can be used in conjunction with the appropriate checklist for that study design (see [www.equator-network.org](http://www.equator-network.org)).
